# X Chromosome Inactivation and *Xist* Evolution in a Rodent Lacking LINE-1 Activity

**DOI:** 10.1371/journal.pone.0006252

**Published:** 2009-07-15

**Authors:** Michael A. Cantrell, Bryan C. Carstens, Holly A. Wichman

**Affiliations:** Department of Biological Sciences, University of Idaho, Moscow, Idaho, United States of America; Louisiana State University, United States of America

## Abstract

Dosage compensation in eutherian mammals occurs by inactivation of one X chromosome in females. Silencing of that X chromosome is initiated by Xist, a large non-coding RNA, whose coating of the chromosome extends in *cis* from the X inactivation center. LINE-1 (L1) retrotransposons have been implicated as possible players for propagation of the Xist signal, but it has remained unclear whether they are essential components. We previously identified a group of South American rodents in which L1 retrotransposition ceased over 8 million years ago and have now determined that at least one species of these rodents, *Oryzomys palustris*, still retains X inactivation. We have also isolated and analyzed the majority of the Xist RNA from *O. palustris* and a sister species retaining L1 activity, *Sigmodon hispidus*, to determine if evolution in these sequences has left signatures that might suggest a critical role for L1 elements in Xist function. Comparison of rates of *Xist* evolution in the two species fails to support L1 involvement, although other explanations are possible. Similarly, comparison of known repeats and potential RNA secondary structures reveals no major differences with the exception of a new repeat in *O. palustris* that has potential to form new secondary structures.

## Introduction

Dosage compensation in eutherian mammals is normally achieved by random inactivation of one of the X chromosomes in females early in development, resulting in silencing of the majority, but not all, of the genes on the inactive X [1]–[3]. This remarkable epigenetic phenomenon is initiated at a locus on the X chromosome called the X-inactivation center (Xic). It involves multiple *cis*- and *trans*-acting factors that work through a number of steps which include counting of the number of X chromosomes, choice of which X chromosome will be inactivated, spreading of the inactivation signal from the Xic along the length of the chromosome, and maintenance of the inactivated state [4], [5]. The *Xist* gene, which is located in the Xic and produces a non-coding RNA larger than 15 kb [6]–[10], is an early, critical player in the initiation and propagation of the inactivation signal. An early step in X chromosome inactivation (XCI) is the *cis* binding of Xist RNA to the chromosome chosen for inactivation by a process which extends the length of that chromosome. The mechanism by which Xist RNA coats the inactive X chromosome and spreads this signal is unknown, but Xist binding precedes other changes in the chromosome including alteration of the methylation and acetylation states of histones, late replication, and methylation of CpG islands for those genes which undergo X inactivation [11]–[13]. Chromosomal localization studies have now shown that Xist coating of the inactive X chromosome leads to formation of a nuclear compartment which excludes the transcription machinery [14]–[16], yet it remains unclear what molecular components are involved in this process.

Gartler and Riggs hypothesized that spreading of the inactivation signal involves some sort of booster elements that facilitate transmission along the chromosome [17]. Studies on the limited spreading of X inactivation in X;autosome translocations [18], [19] and in *Xist* transgenes inserted into autosomes [20] led to suggestions that these booster elements, or way stations, may not be exclusive to the X, but enriched there. In 1998 Lyon hypothesized that LINE-1 (L1) retrotransposons may be the way stations [21], partially because these elements are the most common dispersed repetitive sequences in mammalian genomes, are ubiquitous throughout the mammals, and are at a higher density on X chromosomes than on autosomes [22]–[29].

L1 elements are the most highly represented retroelements in mammalian genomes [22]. Furthermore, in most species that have been examined to date, active L1s from the same species belong to a single lineage and active copies have very high sequence similarity [22]. Even where multiple lineages have been documented, one lineage produces the bulk of new insertions [29]–[31]. Although the high similarity among dispersed L1 copies was not part of the original evidence Lyon considered when she proposed a role for L1s in XCI, such similarity could be an important prerequisite for direct or indirect interaction with Xist.

In the time since Lyon's initial proposal that L1s may function as way stations for propagation of the Xist-mediated inactivation signal, extensive additional information has come to light [32]. Additional species have been shown to preferentially accumulate L1s on the X chromosome, and it has been shown that regions of the X that undergo inactivation tend to have a higher L1 density than those escaping inactivation [24], [26], [33]–[36]. L1s on the inactive X have been shown to be methylated later, and by a different methylase, than those on the active X [37]. Further studies of X;autosome translocations showing incomplete spreading or reduced maintenance of inactivation on autosomal segments have reinforced the idea that there is an inherent difference between the autosomes and the X [38]–[45]. Two groups have shown that L1 densities in X;autosome translocations appear to support the Lyon hypothesis [46], [47], and another group has shown that even though L1 elements appear to be a major factor correlated with X chromosome inactivation, a number of other DNA sequence features may also influence X inactivation [48], [49]. Even on the autosomes, higher L1 densities are correlated with monoallelically expressed genes [50].

Still other results have been interpreted as either not supporting the involvement of L1s in XCI, or suggesting a less critical role [34], [51], [52]. The identification of different types of heterochromatin on the X [53] and of important boundary elements [54] has highlighted suggestions that control of inactivation may occur at both the gene-specific level and the level of chromosomal domains [12], [55].

Yet the recent studies showing that accumulation of Xist on the inactive X chromosome leads to formation of a transcriptionally repressive nuclear compartment, continue to implicate involvement of repetitive sequences in XCI [14], [15]. It has been shown that the compartment initially contains silenced repetitive DNA, with genic DNA only shown to enter the compartment during later silencing of those sequences. This raises the question once again of whether specific types of repeated sequences may be involved.

Even though the process now appears likely to involve cooperative interactions among multiple molecules and multiple regions of the Xist RNA, it is unclear which interactions are direct and which are mediated by other molecules. Similarly, despite large amounts of correlational evidence, the potential role of L1s in this process remains unclear. Their density patterns on chromosomes may reflect their function in X chromosome inactivation or may alternatively be a consequence of their biology and evolutionary history [36]. Even if L1s do play a role in XCI, that role could involve direct interaction with other components of the process, such as Xist, or could be more generally related to their repeated nature, nucleotide composition or other features. The prevalence of L1s in all mammalian genomes makes it difficult to discern whether their presence is a necessary part of the X inactivation process.

If L1 elements are indeed major players in this process, then loss of L1 activity in a species, followed by the mutational decay seen in previously deposited L1 sequences as they mutate over time, should have repercussions for X inactivation. The gradual loss of putative way stations by mutational decay would eventually lead to escape from inactivation for X-linked genes unless there is sufficient compensatory evolution. If interaction between L1s and Xist is direct, a higher rate of evolution of the *Xist* gene would be expected in order to ensure recognition of either way stations that are mutating to greater degeneracy or co-option of different repetitive sequences to serve as way stations. Even if recognition is indirect, increased evolution of intermediate factors might drive compensatory evolution of *Xist*.

We previously identified the first known L1 extinction event in a group of sigmodontine rodents in which L1 activity appears to have ceased about 8.8 million years ago [56], [57]. These rodents have served as a model system to study the effects of L1 elements on genome evolution [58]. In this paper, we have used them to examine the repercussions of loss of L1 activity on X inactivation and *Xist* evolution.

We show here that the L1-inactive species, *Oryzomys palustris*, still exhibits X chromosome inactivation in spite of extinction of L1 activity. We have also isolated the majority of the Xist RNA from *O. palustris* and an L1-active sister species, *Sigmodon hispidus*. We compare these using published *Xist* sequences from the mouse, *Mus musculus*, and the vole, *Microtus arvalis*, as outgroups; we find no statistical support for more rapid evolution of the Xist RNA in *O. palustris* either by analysis of the overall dataset or by sliding window analysis of smaller regions. These results provide no support for the involvement of L1s in X chromosome inactivation, but additional interpretations remain possible.

Previously known tandem repeats within this region of *Xist*, some of which are known to be essential for Xist function, are found here to show no unusual changes, but *O. palustris* has gained a new repeat with potential to form RNA secondary structures. A number of regions of *Xist* are also found to be more highly conserved in all four species examined, implicating specific *Xist* regions for importance in X inactivation.

## Materials and Methods

### Tissues and DNA

Female (accession numbers TK47552 and TK72607) and male (accession no. TK72547) *S. hispidus* tissues were obtained from The Museum at Texas Tech University. Female (KE03 and F2) and male (KE02) *O. palustris* tissues were obtained from Dr. Kent Edmonds (Indiana University, New Albany, IN). Genomic DNA was extracted as previously described [59].

### Isolation of CpG islands and methylation analysis

CpG islands at the 5′ ends of the *Zfx* genes from *O. palustris* and *S. hispidus* were cloned after PCR amplification using previously described primers and amplification conditions [60].

A methylation assay that does not require fresh tissue was employed due to the problems of working with non-laboratory species. This methylation assay used differential cutting of male and female genomic DNA by the restriction enzymes *Hpa*II and *Msp*I, followed by PCR and agarose gel analysis of the CpG islands associated with those genes [61], [62].

### Isolation and sequencing of transcribed regions of the Xist gene

The majority of the region transcribed in the *Xist* gene was isolated from both *O. palustris* and *S. hispidus* by a combination of PCR and RT-PCR in three steps, walking from a 5′ region into overlapping 3′ regions. Degenerate primers were based on previous design considerations [63] and on alignments of previously isolated *Xist* genes. The most 5′ primer was also based on alignment of multiple copies of the A repeat from *Mus musculus* and three *Microtus* species [7], [10]. Sequence obtained from the 3′ part of region 1 for each species was used to design nondegenerate 5′ PCR primers for isolation of region 2, and the same approach was used for region 3. Primer sequences and PCR conditions are in Methods S1. Total RNA was isolated with RNeasy midi kits (Qiagen Corp., Valencia, CA), and cDNA synthesis was done using the Superscript 1^st^ Strand Synthesis System (Invitrogen Corp., Carlsbad, CA). The amplified region 3 sequence from *S. hispidus* is 1,940 bp shorter than the *O. palustris* sequence. This appears to be due to hybridization of the 3′ XT9R PCR primer to an interstitial area showing sequence similarity in both species.

Sequencing was done with an Applied Biosystems 3730 DNA Analyzer. Unless otherwise specified, contig analyses and initial sequence analyses were done using the LASERGENE (DNASTAR, Madison, WI) and Vector NTI (Informax, Bethesda, MD) analysis packages. The cloned *Xist* sequences present between the most 5′ and 3′ primers have GenBank accession numbers GQ201417 (*O. palustris*) and GQ201418 (*S. hispidus)*.

### Alignments, tandem repeat analysis, and phylogenetic analysis

Initial *Xist* alignments done using ClustalW included the following transcribed *Xist* regions from each of these species: bp 1–10,039 of the *O. palustris* sequence; bp 1–10,085 of the *S. hispidus* sequence; bp 621–10,541 from the exons of *Microtus arvalis* (GenBank accession no. AJ310129); and bp 638–12,170 of *Mus musculus* (GenBank accession no. NR_001463). The C repeat from the *M. musculus* sequence is present in only one copy in the other species, so all but one copy of this repeat was removed from the alignment by eliminating bases 3,173–4,678 of the *Mus* sequence. The final alignment of 11,615 characters was finished by manual adjustment (see Alignment S1). For phylogenetic analyses, gapped sites and regions where alignment ambiguity precluded determination of homology were removed, giving rise to a 7,284 character alignment. Repeat analysis was done using the LASERGENE package (DNASTAR, Madison, WI) and Tandem Repeats Finder [64].

To conduct the likelihood-ratio test (LRT) of the molecular clock, a model of sequence evolution was selected using DT-ModSel [65], and this model was verified to fit the data with an absolute goodness-of-fit test [66]. With maximum likelihood as an optimality criterion, Paup* 4.0 b10 [67] was used to estimate the best phylogeny constrained to fit the molecular clock and the best unconstrained phylogeny. The difference in log-Likelihood scores between these phylogenies (lnL_clock_−lnL_unconstrained_) formed the test statistic that was evaluated against the χ^2^ distribution.

Sliding window analyses were done by constructing likelihood trees for either non-overlapping adjacent windows or overlapping windows. The model of sequence evolution selected for the entire gene was used within each window, but parameter values were re-optimized for each of the windows. The command files used for non-overlapping sliding window analyses are in Analyses S1.

## Results

### The Zfx gene shows X chromosome inactivation in females from both Oryzomys palustris and *Sigmodon hispidus*

**Figure 1:**
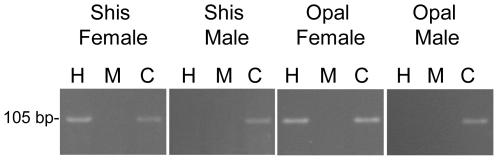
X-inactivation of the Zfx gene from S. hispidus (Shis) and O. palustris (Opal). Genomic DNA from female and male individuals was digested with either *Hpa*II (H, methylation sensitive), *Msp*I (M, methylation insensitive isoschizomer of *Hpa*II), or *Bam*HI (C, control with no *Bam*HI sites in the regions amplified). Subsequent PCR for a portion of the *Zfx* CpG island, followed by gel electrophoresis, resulted in the band seen above only if there was no restriction digestion within the amplified CpG island. The amplification of a CpG island PCR product in females after digestion with *Hpa*II shows that the island contains methylated CpGs, and therefore an inactivated gene. Absence of a product after *Hpa*II digestion in males shows that their CpG island is nonmethylated and therefore capable of activity. *Msp*I cuts the CpG island, serving as a negative control, while *Bam*HI does not cut the CpG island, serving as a positive control.

To address the question of whether X chromosome inactivation can occur in a mammalian species that no longer has active L1 elements, we looked at the methylation status of the CpG island near the 5′ end of the X-linked gene, *Zfx*, in *O. palustris* and a sister sigmodontine species that has retained L1 activity, *S. hispidus*. A portion of the CpG island of the *Zfx* gene from each species was initially cloned and sequenced to confirm its identity, using PCR primers previously shown to amplify this island in other mammalian species. The methylation assay shown in Figure 1 was performed by digesting genomic DNA with either the methylation-sensitive enzyme, *Hpa*II, or the methylation-insensitive isoschizomer, *Msp*I, then PCR amplifying the CpG island. After *Hpa*II digestion and PCR, presence of the predicted band in females (but not in males) from both species shows that *Zfx* undergoes X inactivation in both species.

This result, showing that XCI can still occur more than 8 million years after loss of L1 activity, suggests that highly similar L1 elements are not necessary for propagation of the X-inactivation signal in this species. On the other hand we reasoned that if L1s play a role as ‘way stations’ in XCI by direct interaction with Xist RNA, adaptive evolution of the *Xist* gene in *O. palustris* may have allowed L1s to retain their role in XCI despite the divergence of the extinct L1s in this species. Similarly, if another repetitive sequence replaced that function, the *Xist* gene might also be expected to undergo substantial evolution. This led to examination of the rate of evolution of the majority of the Xist RNA in L1-active and L1-inactive species.

### *O. palustris* Xist RNA appears to be evolving at the same rate as *S. hispidus* Xist RNA

**Figure 2:**
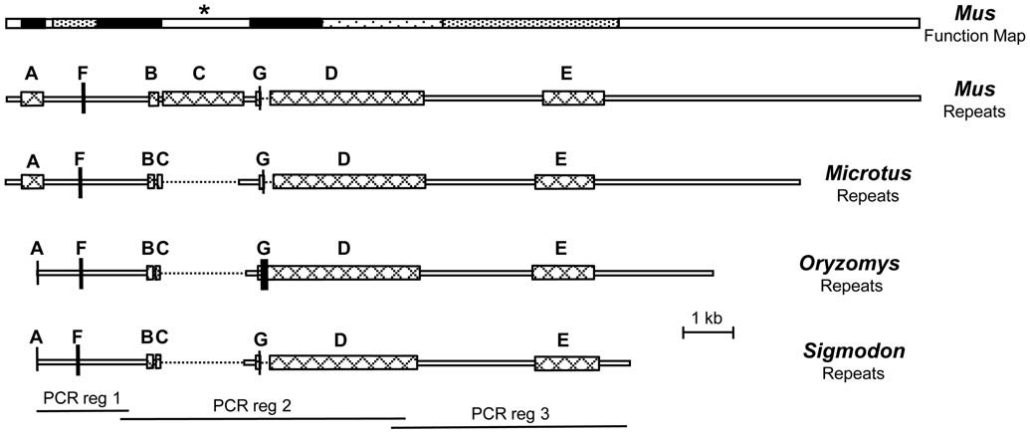
Maps of Xist sequences from four rodent species. Xist RNAs from *Mus musculus*, *Microtus arvalis*, *Oryzomys palustris*, and *Sigmodon hispidus* are aligned, showing the six major previously described tandem repeat regions, A, F, B, C, D, and E, plus the G repeat unique to *O. palustris* (cross hatched boxes or vertical bars). Dotted lines indicate gaps inserted in species which have only a single copy of the C and G repeats. *Mus* and *Microtus* maps show full length RNAs, while the *Oryzomys* and *Sigmodon* maps show the regions isolated in this study. The shaded bar at the top is a functional map summarizing the previously shown relative importance of different regions in *Mus* for Xist silencing and chromosomal localization [68]. The amount of shading in each region of the top bar is proportional to its importance for activity in *Mus*: regions in black showed greatest importance; white regions were found not to be necessary for activity. An asterisk is added above the white bar covering the *Mus* C repeat because other work has suggested it may have an important function for *Mus* Xist [78]. The three regions amplified by PCR are delineated at the bottom. The 5′ primers for amplification of *Xist* from *O. palustris* and *S. hispidus* were located within the A repeat, so only two copies of that repeat from these species were recovered 3′ of the primer bindings sites.

Transcribed *Xist* sequences were isolated from *O. palustris* and *S. hispidus* by a combination of PCR and RT-PCR. This resulted in isolation of the majority of the Xist RNA from both species and covered all of the regions shown by Wutz and coworkers [68] to mediate silencing and Xist localization to the inactive X chromosome in *Mus musculus* (top bar in Figure 2). The regions isolated from both species also include a portion of the 5′ tandem repeat, A, plus all of the other previously known major repeats: F, B, C, D, and E (Figure 2). An alignment that covers approximately 10,050 bp of *Xist* was then modified by removal of gaps and regions showing alignment ambiguity and was used as the basis for phylogenetic analyses. Four species were included in these analyses: the L1-inactive *O. palustris* and its L1-active sister species, *S. hispidus*, plus two L1-active outgroups, *Microtus arvalis* and *Mus musculus*.

**Figure 3:**
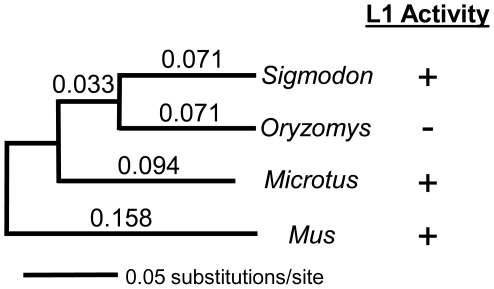
Phylogenetic analysis of Xist sequences from four rodent species. This maximum-likelihood tree was constructed using all ungapped characters that could be unambiguously aligned. Branch lengths are listed. The column on the right indicates whether each species shows L1 activity.

In order to compare rates of *Xist* evolution in the above lineages, we used a likelihood ratio test (LRT) to evaluate the hypothesis that these genes were evolving according to a molecular clock [69]. The null hypothesis was that the rates of evolution did not differ among the four taxa. The HKY+I model of sequence evolution (see Methods Supplement file) was selected and found to have an acceptable absolute fit to the data (*p* = 0.13). When the data were constrained to match the assumptions of the molecular clock, a single optimal phylogeny with −lnL = 21468.81106 was found. The unconstrained phylogeny had a −lnL = 21470.2427 and is shown in Figure 3. As can be seen, the terminal branches for the *O. palustris* and *S. hispidus* sequences are of identical lengths. The difference between the likelihood values for the constrained and unconstrained phylogenies (δ = 2.8632) could not reject the molecular clock at the *P* = 0.95 level of significance (χ^2^ distribution *P*_0.05 (2df)_ = 5.99147). This examination of the dataset *in toto* shows that all branches of the tree appear to be evolving at approximately the same rate, so there is no statistical support for more rapid evolution in *O. palustris* than in *S. hispidus* in spite of the loss of L1 activity.

### Sliding window analysis of contiguous Xist regions

It is possible that specific, small regions of *Xist* are evolving at different rates in each species but that this signal is lost in the total data set, so we also conducted the LRT of the molecular clock using a sliding window approach. Sliding window analyses can be useful in searches for molecular regions important for common functions across a number of species. These would be regions showing reduced evolutionary rates, and thus shorter terminal branches, in all the species examined. Alternatively, a relatively long terminal branch for a particular species, in a window showing short branches in the other species, might be a fingerprint of selection in that species for altered function or loss of function in that region.

Six analyses were done with non-overlapping window sizes of 50, 100, 200, 300, 400, and 500 bp. For each window, we conducted the test both by constraining the topology estimated from the window to the overall species phylogeny, and by search without this constraint. While this approach allowed us to identify particular regions of the *Xist* gene that violated the assumptions of the molecular clock, there was no *a priori* expectation of how many of these regions would result from stochastic processes associated with nucleotide substitution. We generated these expectations by simulating data under the null model (e.g., the phylogeny estimated with the molecular clock enforced) using the model of sequence evolution appropriate for our empirical data. For each window size, we repeated the analysis described above for 1000 simulated data sets.

Table 1 summarizes the results of the above analyses. The top row shows the results obtained when LRTs of the molecular clock were conducted in 14 windows by starting at positions 1–500, then moving in 500 bp steps (e.g., positions 1–500, 501–1000, etc.), until the end of the alignment was reached. No regions were identified where the clock hypothesis was rejected in either the analysis using topologies constrained to the species phylogeny or the analysis with that constraint relaxed. When 400 bp windows were used with constrained tree topologies, one region was found to reject the clock hypothesis, but parametric bootstrapping showed that this number of significant windows is within the expected range (*p* = 0.626). All six window sizes were used in a similar fashion yet none of the 12 analyses showed statistical support for rejection of the molecular clock. The 34 trees arising from the windows showing non-clocklike evolution in Table 1 were visually examined to compare branch lengths, and none of those trees appeared to contain a long *O. palustris* branch. Thus, at these levels of resolution, there is not only no statistical support for an increased rate of evolution within the *O. palustris* sequences, but there is also no suggestive region identified for additional investigation.

**Table 1:** Sliding Window Test of the Molecular Clock.

Window size	Number windows	Number significant windows^a^, constrained topology^b^	P value	Number significant windows^a^, unconstrained topology^c^	P value
500 bp	14	0	1.0	0	1.0
400 bp	18	1	0.626	0	1.0
300 bp	24	1	0.717	1	0.622
200 bp	36	4	0.121	2	0.499
100 bp	72	5	0.701	3	0.435
50 bp	145	10	0.659	7	0.545

a Rejecting the molecular clock at *p* = 0.05 level.

b Constrained to the species topology.

c Not constrained to the species topology.

### Sliding window analysis of overlapping Xist regions

**Figure 4:**
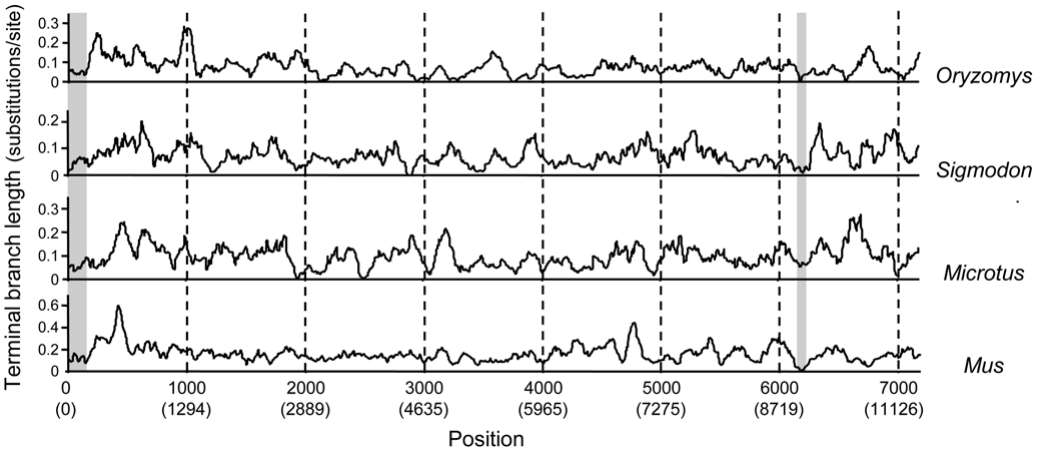
Sliding window analysis of terminal branch lengths of Xist in four rodent species. Likelihood trees were determined for 100 bp windows with a 10 bp slide between each window. The terminal branch length in each window for each species was then plotted. The upper numbers on the X axis indicate the position in the data matrix used for this analysis (see Supplementary Sliding Window file). The lower numbers in parentheses indicate the position in the original alignment before gaps and areas with ambiguous alignment were removed (see Supplementary Alignment file). The shaded areas show two selected regions with short branch lengths in all four species.

To explore the evolution of *Xist* in more detail, we carried out a sliding window analysis similar to the one described above, except with overlapping sliding windows rather than contiguous windows. The results are shown in Figure 4. This approach has increased resolution but precludes statistical analysis because overlapping windows are not independent. For this figure, maximum likelihood trees, unconstrained for the clock but constrained to the species phylogeny, were constructed as described in the previous section from 100 bp windows, but with only a 10 bp slide before moving to the next, partially overlapping window. The relative terminal branch length in each window for each species was then plotted in the figure. We find it striking that there appear to be so few sequence regions with either consistently low or high branch lengths across all four species. This may reflect the lack of sequence conservation which has been reported for *Xist* [9], [10]. Two areas of relatively short branch lengths in all four species are the region at positions 1–200 and the region around position 6200 (grey areas in Figure 4). The 1–200 bp region includes the A repeat, which has been shown to be essential for Xist silencing [68], [70]. The region around 6200 (bp 7806 in the *O. palustris* sequence) is slightly 5′ of the E repeat. It is centered on the exon IV sequence shown to be highly conserved throughout eutherian mammals [71], [72] and predicted to form a stable RNA stem-loop structure involving more than 100 bases [73]. That putative RNA structure appears to be conserved in the *O. palustris* and *S. hispidus* sequences. Interestingly, we see no region that shows a relatively long branch in *O. palustris* coincident with short branches in the other species, and thus no evidence for accelerated evolution of *Xist* in the L1-inactive species.

### Tandem repeats, indels and potential secondary structures in Xist

One of the unusual features of Xist RNA from all species analyzed at this point has been the presence of a surprising number of tandem repeats, which have been proposed to have unknown functions in XCI [7]–[10]. In order to address the question of whether loss of L1 activity is associated with any major change in the amount of tandemly repeated Xist RNA in *O. palustris*, Tandem Repeats Finder was used under multiple stringency settings to determine the total number of bases of tandem repeats in the roughly 10 kb analyzed from each of the four species. Although there were differences between species, *O. palustris* did not show either an unusually high or unusually low amount of tandemly repeated DNA. Similarly, there were no major differences in the number and sizes of indels in the *O. palustris* sequence when compared to the *S. hispidus* sequence, and the overall length of this region (*O. palustris*, 10,039 bases; *S. hispidus*, 10,085 bases) was very similar in each species.

The region analyzed in this study was chosen to include the six major types of tandem repeats that have been previously described in *Xist*. Sequence comparison shows that all six of the repeat regions, the A, B, C, D, E, and F repeat regions found in all other mammals examined to date, are also present in both *S. hispidus* and *O. palustris* (Figure 2). The two copies of the A repeat isolated from both species here retain the same stem and loop sizes shown to be important for Xist silencing in *Mus* [68], suggesting continued functional selection. The C repeat is tandemly duplicated 14 times in *Mus musculus* [7] but present in only one copy in all other species examined.

**Figure 5:**
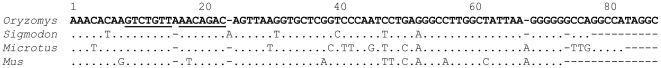
Alignment of the O. palustris G repeat region. The first monomer of the *O. palustris* G repeat, which is repeated four times, is shown in bold. It is aligned with the corresponding single copies in the other three species. Dashes indicate gaps. Periods indicate identity. The first group of 7 underlined bases is a highly conserved sequence separated by one base in *O. palustris* from an inverted copy (also underlined).

A new repeat, G, was found in *O. palustris*, arising from sequence within the D repeat region. The G repeat consists of four copies of an 84 bp unit. The most 5′ copy of this repeat is shown in Figure 5 aligned with the single copies of this region from each of the three other species analyzed in this study. In *O. palustris* this repeat was found to contain a highly conserved 7 base sequence (underlined in Figure 5) separated by one base in each unit from an inverted copy (also underlined). This could give rise to a stem-loop in each of the 4 repeats, or alternatively, pairing between the repeats could produce RNA pseudoknots. It is unclear whether the acquisition of this G repeat in a species which has lost L1 activity has any significance, but is interesting in light of prior suggestions of functional roles for both tandem repeats and secondary structures within Xist. No evidence was found for unusual changes in the other repeats.

## Discussion

If L1s play a major role in propagation of Xist along the inactivated X chromosome [21], loss of L1 activity would certainly have ramifications for XCI. It would seem that XCI must occur in the L1-inactive species, *O. palustris*, since it shows normal XX-XY sex determination, but it was not known until now that this is indeed the case. Our finding of CpG island methylation in a gene on the X in females but not males indicates that XCI does indeed continue to occur in the absence of new L1 deposition indicates that L1 activity per se is not required for XCI, and calls into question any direct role for L1s in X inactivation.

If L1s do play a direct role in XCI, how might XCI continue in the absence of deposition of new L1s? Those elements already present would diverge by accrual of new mutations, and in the absence of retrotransposition of new L1copies, the sequence divergence among L1 elements would increase. What would be the ramifications of such a scenario? There appears to have been no deposition of new L1 elements in *O. palustris* for roughly 8.8 million years [57]. During that time the most recently deposited L1 remnants have diverged from their last active ancestor by 8.6% and from each other by 15.2%. A weak signal in genomic Southern blot analysis and a lack of evidence for X chromosome accumulation in fluorescent *in situ* hybridization provide supporting evidence for the substantial divergence among the fossil L1s remaining in the *O. palustris* genome [56], [57]. The idea that L1s serve as way stations for Xist spread presupposes that at least some part of the L1 sequence is conserved within species. It is possible that the L1s still present in the *O. palustris* genome may not have deteriorated sufficiently by mutational decay for potential way station signals to completely lose function, or this species may have evolved a route for Xist spread that is independent of L1s. If either of these two scenarios is true, then *Xist* sequences might be expected to show a higher rate of evolution in *O. palustris* than in species that have retained L1 activity.

We found that rates of evolution in the *Xist* sequences analyzed here do not differ significantly between *O. palustris* and the three species with L1 activity (*S. hispidus*, *M. arvalis*, *M. musculus*). This result is consistent when the data are analyzed *in toto* as well as when various regions of the gene are analyzed separately with a statistically robust, sliding-window approach. Parametric bootstrapping demonstrated that the numbers of windows where the molecular clock hypothesis was rejected in the empirical data were within the expected range of stochastic variation, and since roughly 1/3 of the sites in this data set are variable, lineages evolving at different rates should be detectable with even the smallest windows used. It is worth noting that the most recently inserted L1s in *O. palustris* and *S. hispidus* are subject to very different evolutionary forces. Neutral substitution and increasing variation among previously deposited L1s predominate in *O. palustris*, while natural selection to maintain an active lineage and very closely related new L1 insertions prevail in *S. hispidus*. The equal rates of Xist evolution in the face of these very different evolutionary processes acting on elements in the L1-inactive and the L1-active species strongly suggests that Xist evolution is independent of L1 evolution.

Even though we found no statistical support for an elevated rate of evolution for *O. palustris* Xist RNA, we cannot unequivocally say that L1s are not involved in XCI. Many models for the interaction of Xist with other molecular components remain possible [74], [75]. The nature of the molecular interactions potentially involving Xist and L1s could greatly affect the expected level of *Xist* evolution. Direct interactions between the two nucleic acids would be likely to elicit a larger evolutionary signal in *Xist* upon loss of L1 activity than a scenario in which the two nucleic acids interact indirectly within a complex. Likewise, it is reasonable to assume that the large functional changes required as a molecular complex evolves to recognize a non-L1 signal might lead to compensatory changes within Xist RNA, even if the two nucleic acids do not interact directly. Our data do not support either of these scenarios. But if the structure of an L1-interacting protein domain is relatively independent of the other molecules involved in the hypothetical complex, then compensatory *Xist* mutation would be less. Under that hypothesis, evolutionary effects on *Xist* might not be observed. If the role for L1s in XCI was not based on direct interaction, but rather on some general feature such as the A-richness of L1s, such role could be maintained in the absence of new L1 deposition for much longer than a role involving direct interactions, e.g., the AT-richness of the *O. palustris* L1s has been maintained since the extinction of L1 activity. An additional possibility is that a region of L1 elements outside of those we have analyzed is important for Xist function, leading to selection for conservation of that L1 region even while loss of L1 activity has lead to neutral mutational decay of the majority of each element.

It also remains possible that the approach used here would not detect small Xist regions altered in a molecular complex that includes L1s. High resolution analyses of these data, such as the one shown in Figure 4, with *Xist* sequences from additional species may allow researchers to pinpoint other slowly evolving regions that may be important for general *Xist* function, and perhaps to identify candidate regions for L1 involvement in XCI.

It is interesting that the sliding window analyses done here detected the previously known conservation of both the A repeat and the exon IV likely stem structure region. This result suggests continued functional importance for these regions in all four species, yet in spite of this sensitivity, no candidate regions were identified for possible L1 mediated function.

An unusual aspect of all published Xist RNAs is their high level of tandem repeats, some of which have been shown to be functionally important, possibly working in a cooperative fashion [9]. The six major repeat regions previously identified were found to be present in the sequences we have analyzed in this study. Their conservation, plus the retention of likely secondary structure in the A repeat, suggests that no major changes have occurred with respect to these repeats. On the other hand, the acquisition of the new G repeat in *O. palustris* is noteworthy. Its potential for the formation of additional RNA secondary structures should be considered in terms of both general *Xist* function and a potential role in a species that has lost L1 activity.

We have explored the possibility that a new family of repetitive sequences may have arisen to high copy numbers in *O. palustris* to either replace putative functions of L1 elements or to fill the niche vacated by loss of L1 activity. We found a family of endogenous retroviruses, the mysTR family, which are present at unprecedented copy numbers of approximately 10,000 relatively closely related elements in this species [58]. However, it remains unclear at this point whether the mysTR family has any relevance to L1 extinction. No obvious difference in densities of mysTR elements on the X versus the autosomes in *O. palustris* was observed, and there is no evidence from the current study that *Xist* underwent rapid evolution to recognize mysTR as an alternate way station. Any correlation of mysTR amplification with loss of L1 activity may be clarified by our ongoing study of this family in a number of rodent species related to the ones described here.

We have also addressed the question of whether L1s might be involved in XCI by looking at L1 activity in a mammal which has lost the need for dosage compensation, the Ryukyu spiny rat, *Tokudaia osimensis*, which has an XO karyotype in both males and females. We reasoned that this karyotype might have evolved as an alternate pathway for dosage compensation after loss of L1 activity. However, we found that L1s are still active in this species and have continued to accumulate at a higher density on the X than on the autosomes [36]. These results support the idea that the higher density of L1s on the X may be due to reduced recombinational removal of L1s on the X, relative to the autosomes [28], [76].

The most parsimonious interpretation for the results presented in this study and in the *Tokudaia* study described above is that L1 elements are not involved in propagation of X inactivation. It remains possible, however, that the general rate of *Xist* evolution is so high [10], or the region of way station recognition is so small, that adaptation in *O. palustris* cannot be detected by these methods. A critically altered region in Xist might also be outside of the area we have analyzed, even though this area includes all the regions that have been shown to be important for silencing and localization [68], [77]. It is also possible that there has not yet been sufficient mutational decay of previously deposited L1s in *O. palustris* to elicit obvious compensatory evolution from homologous *Xist* sequences or that the new G repeat is the result of compensatory evolution in the presence of deteriorating L1 elements.

It is now clear that X inactivation can continue to occur in the absence of new L1 deposition for millions of years. The current study, like so many others aimed at testing the role of L1s in XCI, does not show unequivocally whether they are involved. However, in light of this study and recent work of others, perhaps it is time to consider that even if L1s facilitate XCI in some way; their role might be more complex than that of way stations for the spread of the inactivation signal. Greater progress might be made by focusing on X inactivation models that incorporate the increasing evidence for cooperation between molecules involved in the process, as well as by considering alternative explanations for accumulation of L1s on the X.

## Supporting Information

Methods S1 PCR primers and methods for characterization of the Xist gene
(0.03 MB DOC)

Alignment S1 XIST alignment
(0.08 MB TXT)

Analysis S1 Commands for sliding window analysis of Xist. Areas of ambiguous alignment and gapped sites are excluded from 4the alignment. See Alignment S1 for the complete alignment.
(0.06 MB TXT)

## References

[1] Valley CM, Willard HF (2006). Genomic and epigenomic approaches to the study of X chromosome inactivation.. Curr Opin Genet Dev.

[2] Heard E, Clerc P, Avner P (1997). X-chromosome inactivation in mammals.. Annu Rev Genet.

[3] Straub T, Becker PB (2007). Dosage compensation: the beginning and end of generalization.. Nat Rev Genet.

[4] Chang SC, Tucker T, Thorogood NP, Brown CJ (2006). Mechanisms of X-chromosome inactivation.. Front Biosci.

[5] Heard E, Disteche CM (2006). Dosage compensation in mammals: fine-tuning the expression of the X chromosome.. Genes Dev.

[6] Borsani G, Tonlorenzi R, Simmler MC, Dandolo L, Arnaud D (1991). Characterization of a murine gene expressed from the inactive X chromosome.. Nature.

[7] Brockdorff N, Ashworth A, Kay GF, McCabe VM, Norris DP (1992). The product of the mouse *Xist* gene is a 15 kb inactive X-specific transcript containing no conserved ORF and located in the nucleus.. Cell.

[8] Brown CJ, Hendrich BD, Rupert JL, Lafreniere RG, Xing Y (1992). The human *XIST* gene: analysis of a 17 kb inactive X-specific RNA that contains conserved repeats and is highly localized within the nucleus.. Cell.

[9] Plath K, Mlynarczyk-Evans S, Nusinow DA, Panning B (2002). *Xist* RNA and the mechanism of X chromosome inactivation.. Annu Rev Genet.

[10] Nesterova TB, Slobodyanyuk SY, Elisaphenko EA, Shevchenko AI, Johnston C (2001). Characterization of the genomic *Xist* locus in rodents reveals conservation of overall gene structure and tandem repeats but rapid evolution of unique sequence.. Genome Res.

[11] Heard E (2004). Recent advances in X-chromosome inactivation.. Curr Opin Cell Biol.

[12] Chow JC, Yen Z, Ziesche SM, Brown CJ (2005). Silencing of the mammalian X chromosome.. Annu Rev Genomics Hum Genet.

[13] Salstrom JL (2007). X-inactivation and the dynamic maintenance of gene silencing.. Mol Genet Metab.

[14] Chaumeil J, Le Baccon P, Wutz A, Heard E (2006). A novel role for Xist RNA in the formation of a repressive nuclear compartment into which genes are recruited when silenced.. Genes Dev.

[15] Clemson CM, Hall LL, Byron M, McNeil J, Lawrence JB (2006). The X chromosome is organized into a gene-rich outer rim and an internal core containing silenced nongenic sequences.. Proc Natl Acad Sci USA.

[16] Matarazzo MR, Cerase A, D'Esposito M (2008). Building up the inactive X chromosome.. Biol Cell.

[17] Gartler SM, Riggs AD (1983). Mammalian X-chromosome inactivation.. Annu Rev Genet.

[18] Russell LB, Montgomery CS (1970). Comparative studies on X-autosome translocations in the mouse. II.Inactivation of autosomal loci, segregation, and mapping of autosomal breakpoints in five T (X;1) S.. Genetics.

[19] Rastan S (1983). Non-random X-chromosome inactivation in mouse X-autosome translocation embryos–location of the inactivation centre.. J Embryol Exp Morphol.

[20] Lee JT, Jaenisch R (1997). Long-range cis effects of ectopic X-inactivation centres on a mouse autosome.. Nature.

[21] Lyon MF (1998). X-chromosome inactivation: a repeat hypothesis.. Cytogenet Cell Genet.

[22] Furano AV (2000). The biological properties and evolutionary dynamics of mammalian LINE-1 retrotransposons.. Prog Nucleic Acid Res Mol Biol.

[23] Baker RJ, Kass DH (1994). Comparison of chromosomal distribution of a retroposon (LINE) and a retrovirus-like element mys in *Peromyscus maniculatus* and *P. leucopus*.. Chromosome Res.

[24] Waters PD, Dobigny G, Pardini AT, Robinson TJ (2004). LINE-1 distribution in Afrotheria and Xenarthra: implications for understanding the evolution of LINE-1 in eutherian genomes.. Chromosoma.

[25] Korenberg JR, Rykowski MC (1988). Human genome organization: *Alu*, lines, and the molecular structure of metaphase chromosome bands.. Cell.

[26] Parish DA, Vise P, Wichman HA, Bull JJ, Baker RJ (2002). Distribution of LINEs and other repetitive elements in the karyotype of the bat *Carollia*: implications for X-chromosome inactivation.. Cytogenet Genome Res.

[27] Boyle AL, Ballard SG, Ward DC (1990). Differential distribution of long and short interspersed element sequences in the mouse genome: chromosome karyotyping by fluorescence *in situ* hybridization.. Proc Natl Acad Sci U S A.

[28] Wichman HA, Van den Bussche RA, Hamilton MJ, Baker RJ (1992). Transposable elements and the evolution of genome organization in mammals.. Genetica.

[29] Casavant NC, Lee RN, Sherman AN, Wichman HA (1998). Molecular evolution of two lineages of L1 (LINE-1) retrotransposons in the California mouse, *Peromyscus californicus*.. Genetics.

[30] Casavant NC, Sherman AN, Wichman HA (1996). Two persistent LINE-1 lineages in *Peromyscus* have unequal rates of evolution.. Genetics.

[31] Casavant NC, Hardies SC (1994). The dynamics of murine LINE-1 subfamily amplification.. J Mol Biol.

[32] Lyon MF (2005). No longer ‘all-or-none’.. Eur J Hum Genet.

[33] Bailey JA, Carrel L, Chakravarti A, Eichler EE (2000). Molecular evidence for a relationship between LINE-1 elements and X chromosome inactivation: the Lyon repeat hypothesis.. Proc Natl Acad Sci USA.

[34] Carrel L, Willard HF (2005). X-inactivation profile reveals extensive variability in X-linked gene expression in females.. Nature.

[35] Ross MT, Grafham DV, Coffey AJ, Scherer S, McLay K (2005). The DNA sequence of the human X chromosome.. Nature.

[36] Scott L, Kuroiwa A, Matsuda M, Wichman HA (2006). X accumulation of LINE-1 retrotransposons in *Tokudaia osimensis*, a spiny rat with the karyotype XO.. Cytogenet Genome Res.

[37] Hansen RS (2003). X inactivation-specific methylation of LINE-1 elements by DNMT3B: implications for the Lyon repeat hypothesis.. Hum Mol Genet.

[38] Sharp A, Robinson DO, Jacobs P (2001). Absence of correlation between late-replication and spreading of X inactivation in an X;autosome translocation.. Hum Genet.

[39] Hall LL, Clemson CM, Byron M, Wydner K, Lawrence JB (2002). Unbalanced X; autosome translocations provide evidence for sequence specificity in the association of XIST RNA with chromatin.. Hum Mol Genet.

[40] White WM, Willard HF, Van Dyke DL, Wolff DJ (1998). The spreading of X inactivation into autosomal material of an x;autosome translocation: evidence for a difference between autosomal and X-chromosomal DNA.. Am J Hum Genet.

[41] Keohane AM, Barlow AL, Waters J, Bourn D, Turner BM (1999). H4 acetylation, XIST RNA and replication timing are coincident and define x;autosome boundaries in two abnormal X chromosomes.. Hum Mol Genet.

[42] Solari AJ, Rahn IM, Ferreyra ME, Carballo MA (2001). The behavior of sex chromosomes in two human X-autosome translocations: failure of extensive X-inactivation spreading.. Biocell.

[43] Noronha RCR, Nagamachi CY, Pieczarka JC, Marques-Agular S, Barros RMS (2001). Sex-autosome translocations: meiotic behaviour suggests an inactivation block with permanence of autosomal gene activity in Phyllostomid bats.. Caryologia.

[44] Duthie SM, Nesterova TB, Formstone EJ, Keohane AM, Turner BM (1999). *Xist* RNA exhibits a banded localization on the inactive X chromosome and is excluded from autosomal material in *cis*.. Hum Mol Genet.

[45] Sharp AJ, Spotswood HT, Robinson DO, Turner BM, Jacobs PA (2002). Molecular and cytogenetic analysis of the spreading of X inactivation in X;autosome translocations.. Hum Mol Genet.

[46] Dobigny G, Ozouf-Costaz C, Bonillo C, Volobouev V (2004). Viability of X-autosome translocations in mammals: an epigenomic hypothesis from a rodent case-study.. Chromosoma.

[47] Popova BC, Tada T, Takagi N, Brockdorff N, Nesterova TB (2006). Attenuated spread of X-inactivation in an X;autosome translocation.. Proc Natl Acad Sci USA.

[48] Wang Z, Willard HF, Mukherjee S, Furey TS (2006). Evidence of influence of genomic DNA sequence on human X chromosome inactivation.. PLoS Comput Biol.

[49] Carrel L, Park C, Tyekucheva S, Dunn J, Chiaromonte F (2006). Genomic environment predicts expression patterns on the human inactive X chromosome.. PLoS Genet.

[50] Allen E, Horvath S, Tong F, Kraft P, Spiteri E (2003). High concentrations of long interspersed nuclear element sequence distinguish monoallelically expressed genes.. Proc Natl Acad Sci USA.

[51] Chureau C, Prissette M, Bourdet A, Barbe V, Cattolico L (2002). Comparative sequence analysis of the X-inactivation center region in mouse, human, and bovine.. Genome Res.

[52] Ke X, Collins A (2003). CpG islands in human X-inactivation.. Ann Hum Genet.

[53] Chadwick BP, Willard HF (2004). Multiple spatially distinct types of facultative heterochromatin on the human inactive X chromosome.. Proc Natl Acad Sci USA.

[54] Filippova GN, Cheng MK, Moore JM, Truong JP, Hu YJ (2005). Boundaries between chromosomal domains of X inactivation and escape bind CTCF and lack CpG methylation during early development.. Dev Cell.

[55] Tsuchiya KD, Greally JM, Yi Y, Noel KP, Truong JP (2004). Comparative sequence and x-inactivation analyses of a domain of escape in human xp11.2 and the conserved segment in mouse.. Genome Res.

[56] Casavant NC, Scott L, Cantrell MA, Wiggins LE, Baker RJ (2000). The end of the LINE?: lack of recent L1 activity in a group of South American rodents.. Genetics.

[57] Grahn RA, Rinehart TA, Cantrell MA, Wichman HA (2005). Extinction of LINE-1 activity coincident with a major mammalian radiation in rodents.. Cytogenet Genome Res.

[58] Cantrell MA, Ederer MM, Erickson IK, Swier VJ, Baker RJ (2005). MysTR: an Endogenous Retrovirus Family in Mammals Undergoing Recent Amplifications to Unprecedented Copy Numbers.. J Virol.

[59] Longmire JL, Lewis AK, Brown NC, Buckingham JM, Clark LM (1988). Isolation and molecular characterization of a highly polymorphic centromeric tandem repeat in the family Falconidae.. Genomics.

[60] Luoh SW, Jegalian K, Lee A, Chen EY, Ridley A (1995). CpG islands in human ZFX and ZFY and mouse Zfx genes: sequence similarities and methylation differences.. Genomics.

[61] Jegalian K, Page DC (1998). A proposed path by which genes common to mammalian X and Y chromosomes evolve to become X inactivated.. Nature.

[62] Tribioli C, Tamanini F, Patrosso C, Milanesi L, Villa A (1992). Methylation and sequence analysis around EagI sites: identification of 28 new CpG islands in XQ24-XQ28.. Nucleic Acids Res.

[63] Cantrell MA, Grahn RA, Scott L, Wichman HA (2000). Isolation of markers from recently transposed LINE-1 retrotransposons.. Biotechniques.

[64] Benson G (1999). Tandem repeats finder: a program to analyze DNA sequences.. Nucleic Acids Res.

[65] Minin V, Abdo Z, Joyce P, Sullivan J (2003). Performance-based selection of likelihood models for phylogeny estimation.. Syst Biol.

[66] Goldman N (1993). Statistical tests of models of DNA substitution.. J Mol Evol.

[67] Swofford DL (2002). PAUP*. Phylogenetic Analysis Using Parsimony (*and Other Methods). Version 4.

[68] Wutz A, Rasmussen TP, Jaenisch R (2002). Chromosomal silencing and localization are mediated by different domains of *Xist* RNA.. Nat Genet.

[69] Felsenstein J (1988). Phylogenies from molecular sequences: inference and reliability.. Annu Rev Genet.

[70] Hoki Y, Kimura N, Kanbayashi M, Amakawa Y, Ohhata T (2009). A proximal conserved repeat in the Xist gene is essential as a genomic element for X-inactivation in mouse.. Development.

[71] Hore TA, Koina E, Wakefield MJ, Marshall Graves JA (2007). The region homologous to the X-chromosome inactivation centre has been disrupted in marsupial and monotreme mammals.. Chromosome Res.

[72] Duret L, Chureau C, Samain S, Weissenbach J, Avner P (2006). The Xist RNA gene evolved in eutherians by pseudogenization of a protein-coding gene.. Science.

[73] Caparros ML, Alexiou M, Webster Z, Brockdorff N (2002). Functional analysis of the highly conserved exon IV of XIST RNA.. Cytogenet Genome Res.

[74] Pauler FM, Koerner MV, Barlow DP (2007). Silencing by imprinted noncoding RNAs: is transcription the answer?. Trends Genet.

[75] Wutz A (2007). Xist function: bridging chromatin and stem cells.. Trends Genet.

[76] Baker RJ, Wichman HA (1990). Retrotransposon mys is concentrated on the sex chromosomes: implications for copy number containment.. Evolution.

[77] Chow JC, Hall LL, Baldry SE, Thorogood NP, Lawrence JB (2007). Inducible XIST-dependent X-chromosome inactivation in human somatic cells is reversible.. Proc Natl Acad Sci USA.

[78] Beletskii A, Hong YK, Pehrson J, Egholm M, Strauss WM (2001). PNA interference mapping demonstrates functional domains in the noncoding RNA *Xist*.. Proc Natl Acad Sci USA.

