# Supplementary material for: X Chromosome Inactivation and Xist Evolution in a Rodent Lacking LINE-1 Activity
Source: PLoS One. 2009 Jul 15;4(7):e6252. doi: 10.1371/journal.pone.0006252 (PMC2705805; doi:10.1371/journal.pone.0006252)
Supplement: Methods S1 — PCR primers and methods for characterization of the Xist gene (0.03 MB DOC) [file pone.0006252.s001.doc]

**Methods S1.** PCR primers and methods for characterization of the *Xist* gene.

*Isolation and sequencing of transcribed regions:*

PCRs were performed on a Mastercycler gradient machine (Eppendorf Corp., Hamburg) using the Applied Biosystems (Foster City, CA) extra-long PCR system in a total volume of 50 µl using 100 pmol of each primer; 200 ng of genomic DNA or 5 ng of total RNA, and 0.8-1.5 mM magnesium acetate. Each PCR reaction was divided into three cycling sets. The first set of cycles employed touchdown PCR with annealing temperatures related to the Tm of the target site homology regions within the primers. The second set of cycles used higher annealing temperatures related to the Tm of the full primer lengths, including 5' clamp sequences; and the third set of cycles added increasing elongation times.

Region 1 from O. palustris was amplified with the following parameters: Hot starts were performed by combining all components except the genomic DNA and the polymerase, heating to 70°C, then adding these final two components before cycling. Cycling parameters were as follows. Touchdown PCR cycles: 70°C hot-start for 5 min; 94°C denaturation for 1.5 min; then touchdown PCR by 8 cycles of 94°C denaturation for 15 s, 64°C annealing for 30 s (and 1°C temperature reduction at each subsequent cycle), 70°C elongation for 2 min; followed by 3 cycles of 94°C for 15 sec., 56°C for 30 sec., 70°C for 2 min. PCR continued with higher annealing temperatures: 9 cycles of 94°C for 15 s, 62°C for 30 s, 70°C for 2 min. Cycling was then continued by 12 additional cycles using the same conditions but with elongation times increased by 15 s for each cycle and a final 72°C extension for 10 min. Amplification of region 1 from S. hispidus was as above but with the annealing temperatures 6°C lower during the first 11 cycles. All extension times were increased by 4 min. for regions 2 and 3, and annealing temperatures were adjusted for different primer Tm's. Regions 1 and 2 are within the first exon of previously isolated Xist genes.

*PCR primers:*

Primer names containing an F signify forward primers and those containing an R, reverse primers. The underlined parts of primers indicate the Xist homology regions.

Primers for region 1 in both *O. palustris* and *S. hispidus*:

XT3F: 5'-CCTTATCTAGACGGGGYIIYGGATABCTG-3'

XT3R: 5'-ATGGTACCTCCAGCAWTAGYCANGGGGTAG-3'

Primers for region 2 in *S. hispidus*:

XT4FS: 5'-CCC TTA TCT AGA GCC CGC ACT GCG ATC TCA AG-3'

XT4R: 5'-ATG GTA CCA RGR TGG CAA TCC ARC TCC CTG-3'

Primers for region 2 in *O. palustris*:

XT4RC: 5'-ARG RTG GCA ATC CAR CTC CCT G-3' (used as primer for first strand cDNA synthesis.)

XT4FO: 5'-CCC TTA TCT AGA GCC GGC ACT GCC ATC TCA AG-3'

XT4R: 5'-ATG GTA CCA RGR TGG CAA TCC ARC TCC CTG-3'

Primers for region 3 in *S. hispidus*:

XT23FSB: 5'-GCC TGC CTT TAT TAA TGC CTT AC-3'

XT9R: 5'-CCC TTA TCT AGA AWT TGT TAY ATT TAT ATG GT-3' (Used for both cDNA synthesis and PCR)

Primers for region 3 in *O. palustris*:

XT21FO: 5'-CAA AAC TAG GTG GAT GAA TCT TCG TTC-3'

XT11R: 5'-TGG TAC CTA TGG CAR YTT ACA RIT TAT AAA TTA-3' (Used for both cDNA synthesis and PCR.)

*Sequencing:*

Sequencing was carried out on an Applied Biosystems 3730 DNA Analyzer. Unless otherwise specified, contig analyses and sequence analyses were done using the LASERGENE (DNASTAR, Madison, WI) and Vector NTI (Informax, Bethesda, MD) analysis packages.

*Phylogenetic analysis:*

The model parameters for the HKY + I model of sequence evolution used for examination of the data set in toto were: base frequencies, A = 0.2547, C = 0.21609, G = 0.21188, T = 0.31733; Ti/Tv ratio = 1.98622; pinv = 0.18824. Sliding window analyses were done with a range of window sizes because larger windows give the additional power that comes with analysis of more data at the expense of reduced resolution, while smaller windows give greater resolution but with less information in each window.
